# 1-[2-(1*H*-Benzimidazol-2-yl)eth­yl]-1*H*-1,2,3-benzotriazole

**DOI:** 10.1107/S1600536811045442

**Published:** 2011-11-05

**Authors:** Zhong Zhang, Wei Lu, Difeng Wu

**Affiliations:** a College of Chemistry and Chemical Engineering, Guangxi Normal University, Yucai Road 15, Guilin 541004, People’s Republic of China

## Abstract

In the title compound, C_15_H_13_N_5_, the N-containing heterocycles are linked by an ethyl­ene spacer in a *gauche* conformation, the N—C—C—C torsion angle along the linker being 60.1 (3)°. The dihedral angle between the terminal benzotriazole and benzimidazole rings is 39.02 (6)°. In the crystal, adjacent mol­ecules are connected by N—H⋯N hydrogen bonds, forming an infinite chain along the *c* axis. π–π stacking inter­actions [centroid–centroid distance = 3.8772 (7) Å] between the benzotriazole rings of neighbouring chains extend these chains into a supra­molecular sheet in the *bc* plane. Weak inter­molecular C—H⋯N inter­actions further stabilize the crystal structure.

## Related literature

For the synthesis and anti­viral activity of bis-heterocyclcic compounds containing both benzotriazole and benzimidazole, see: Pagani & Sparatore (1965 ▶); Paglietti *et al.* (1975 ▶); Katritzky *et al.* (1996 ▶); Yu *et al.* (2003 ▶); Tonelli *et al.* (2008 ▶). For the crystal structure of 1-(benzimidazol-2-ylmeth­yl)-1*H*-benzotriazole, see: Liu *et al.* (2007 ▶).
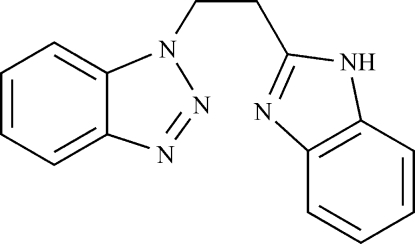


## Experimental

### 

#### Crystal data

C_15_H_13_N_5_
*M* *_r_* = 263.30
Monoclinic, 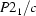

*a* = 6.3510 (13) Å
*b* = 20.830 (4) Å
*c* = 9.901 (2) Å
β = 96.78 (3)°
*V* = 1300.7 (5) Å^3^
*Z* = 4
Mo *K*α radiation
μ = 0.09 mm^−1^
*T* = 294 K
0.37 × 0.32 × 0.26 mm

#### Data collection

Bruker APEX CCD area-detector diffractometer
Absorption correction: multi-scan (*SADABS*; Bruker, 2001) ▶ *T*_min_ = 0.969, *T*_max_ = 0.978
10985 measured reflections
2290 independent reflections
1608 reflections with *I* > 2σ(*I*)
*R*_int_ = 0.071

#### Refinement

*R*[*F* ^2^ > 2σ(*F* ^2^)] = 0.063
*wR*(*F* ^2^) = 0.125
*S* = 1.01
2290 reflections
181 parameters
H-atom parameters constrained
Δρ_max_ = 0.18 e Å^−3^
Δρ_min_ = −0.21 e Å^−3^

### 

Data collection: *SMART* (Bruker, 2007 ▶); cell refinement: *SAINT* (Bruker, 2007 ▶); data reduction: *SAINT*; program(s) used to solve structure: *SHELXTL* (Sheldrick, 2008 ▶); program(s) used to refine structure: *SHELXTL*; molecular graphics: *SHELXTL* and *DIAMOND* (Brandenburg, 1999 ▶); software used to prepare material for publication: *SHELXTL*.

## Supplementary Material

Crystal structure: contains datablock(s) I, global. DOI: 10.1107/S1600536811045442/zj2029sup1.cif

Structure factors: contains datablock(s) I. DOI: 10.1107/S1600536811045442/zj2029Isup2.hkl

Supplementary material file. DOI: 10.1107/S1600536811045442/zj2029Isup3.cml

Additional supplementary materials:  crystallographic information; 3D view; checkCIF report

## Figures and Tables

**Table 1:** Hydrogen-bond geometry (Å, °)

*D*—H⋯*A*	*D*—H	H⋯*A*	*D*⋯*A*	*D*—H⋯*A*
N4—H14⋯N5^i^	0.84	2.04	2.855 (3)	162
C1—H1⋯N3^ii^	0.93	2.54	3.456 (3)	167

Symmetry codes: (i) 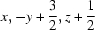; (ii) 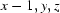.

**Fig. 1.**
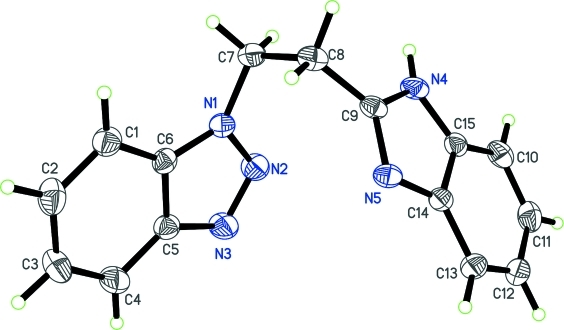
The molecular structure of the title compound (I), showing the atom labeling scheme and 30% displacement ellipsoids.

**Fig. 2.**
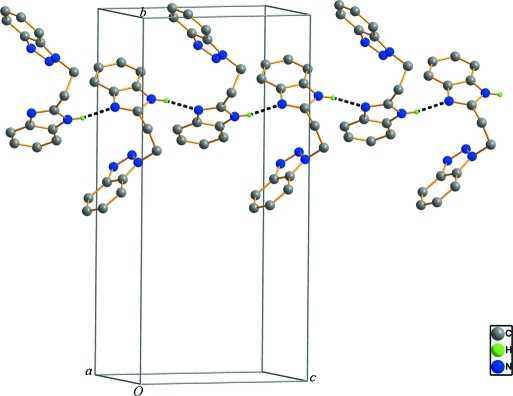
The crystal packing of the title compound (I), showing the one-dimensional hydrogen-bonding chain. Hydrogen bonds are shown as black dashed lines.

**Fig. 3.**
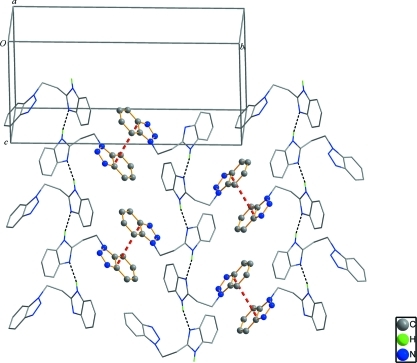
A view of the two-dimensional supramolecular sheet constructed from one-dimensional chains viaπ—π stacking of heterocyclic rings. π—π stacking interactions are represented by red dashed lines.

## supplementary crystallographic information

### Comment

A family of asymmetric bis-heterocycle compounds comprising both benzotriazole and benzimidazole entities have been prepared (Pagani *et al.* 1965; Paglietti *et al.* 1975; Katritzky *et al.* 1996) and some of them exhibit potent antiviral activity (Yu *et al.* 2003; Tonelli *et al.* 2008), but the structure-function relationship of these novel potential drugs is still a research focus. The structural determination of 1-(Benzimidazol-2-ylmethyl)-1*H*-benzotriazole has been fulfilled (Liu *et al.* 2007). In this paper, the crystal structure of its analogue 1-(2-(1*H*-Benzimidazol-2-yl)ethyl)-1*H*-benzotriazole was reported. The molecular structure of the title compound, (I), is depicted in Fig. 1. In the molecule, benzotriazole and benzimidazole rings are arranged in a *gauche* conformation about the ethylidene linkage, as described by the N1—C7—C8—C9 torsion angle of 60.1 (3) °. Both heterocyclic rings are planar and the dihedral angle between them is 39.02 (6) °. In the crystal packing, adjacent molecules are self-assembled through intermolecular N4—H14···N5^i^ hydrogen bonds (Table 1) between benzimidazole groups into an infinite one-dimensional non-linear chain along the *c* axis (Fig. 2), which is further held together *via* face-to-face π—π stacking [centriod-centriod distance = 3.8772 (7) Å, symmetry code: -*x*, 1 - *y*,2 - *z*] between the benzotriazole groups coming from neighbouring non-linear chains resulting in a two-dimensional supramolecular layer parallel to the *bc* plane (Fig. 3). Weak C1—H1···N3^ii^ hydrogen bonds (Table 1) exist between the layers contributing to the construction of the three-dimensional network.

### Experimental

The mixture of 3-(1*H*-benzotriazole-1-yl)-propionic acid (3.8 g, 0.02 mol) and ο-phenylenediamine (3.2 g, 0.03 mol) were dissolved in 2 mol/*L* HCl (10 ml) and refluxed for 10 h to yield a brown solution. After cooling, the solution was filtered to remove the unreacted ο-phenylenediamine. Concentrated NH_3_.H_2_O was added to the resulting filtrate continuously until the gray solid was precipitated completely. The gray solid was redissolved in a small amount of CH_3_OH at room temperature. Two weeks ago, colorless block-like crystals of (I) suitable for X-ray diffraction analysis were obtained. (yield 47%) Elemental analysis found: C 68.69; H 5.32; N 26.64%; calculated for C_15_H_13_N_5_: C 68.42; H 4.98; N 26.60%.

### Refinement

All H atoms were positioned geometrically (C—H = 0.93–0.97 Å and N—H = 0.84 Å) and refined as riding atoms, with *U*_iso_(H) = 1.2 times *U*_eq_(C) or 1.5 times *U*_eq_(N).

### Crystal data

**Table:**

C_15_H_13_N_5_	*F*(000) = 552
*M**_r_* = 263.30	*D*_x_ = 1.345 Mg m^−^^3^
Monoclinic, *P*2_1_/*c*	Mo *K*α radiation, λ = 0.71073 Å
Hall symbol: -P 2ybc	Cell parameters from 10162 reflections
*a* = 6.3510 (13) Å	θ = 3.2–27.8°
*b* = 20.830 (4) Å	µ = 0.09 mm^−^^1^
*c* = 9.901 (2) Å	*T* = 294 K
β = 96.78 (3)°	Block, colourless
*V* = 1300.7 (5) Å^3^	0.37 × 0.32 × 0.26 mm
*Z* = 4	

### Data collection

**Table:**

Bruker APEX CCD area-detector diffractometer	2290 independent reflections
Radiation source: fine-focus sealed tube	1608 reflections with *I* > 2σ(*I*)
graphite	*R*_int_ = 0.071
phi and ω scans	θ_max_ = 25.0°, θ_min_ = 3.2°
Absorption correction: multi-scan (*SADABS*; Bruker, 2001)	*h* = −7→7
*T*_min_ = 0.969, *T*_max_ = 0.978	*k* = −24→24
10985 measured reflections	*l* = −11→11

### Refinement

**Table:**

Refinement on *F*^2^	Primary atom site location: structure-invariant direct methods
Least-squares matrix: full	Secondary atom site location: difference Fourier map
*R*[*F*^2^ > 2σ(*F*^2^)] = 0.063	Hydrogen site location: inferred from neighbouring sites
*wR*(*F*^2^) = 0.125	H-atom parameters constrained
*S* = 1.01	*w* = 1/[σ^2^(*F*_o_^2^) + (0.040*P*)^2^ + 0.8*P*] where *P* = (*F*_o_^2^ + 2*F*_c_^2^)/3
2290 reflections	(Δ/σ)_max_ = 0.001
181 parameters	Δρ_max_ = 0.18 e Å^−^^3^
0 restraints	Δρ_min_ = −0.21 e Å^−^^3^

### Special details

**Table:**

Refinement. Refinement of *F*^2^ against ALL reflections. The weighted *R*-factor *wR* and goodness of fit *S* are based on *F*^2^, conventional *R*-factors *R* are based on *F*, with *F* set to zero for negative *F*^2^. The threshold expression of *F*^2^ > σ(*F*^2^) is used only for calculating *R*-factors(gt) *etc*. and is not relevant to the choice of reflections for refinement. *R*-factors based on *F*^2^ are statistically about twice as large as those based on *F*, and *R*- factors based on ALL data will be even larger.

### Fractional atomic coordinates and isotropic or equivalent isotropic displacement parameters (Å^2^)

**Table:**

	*x*	*y*	*z*	*U*_iso_*/*U*_eq_	
C1	−0.2105 (4)	0.96993 (13)	0.3185 (3)	0.0476 (7)	
H1	−0.3426	0.9620	0.3468	0.057*	
C2	−0.1838 (5)	1.01290 (14)	0.2171 (3)	0.0573 (8)	
H2	−0.3016	1.0346	0.1748	0.069*	
C3	0.0144 (5)	1.02507 (15)	0.1755 (3)	0.0567 (8)	
H3	0.0252	1.0549	0.1068	0.068*	
C4	0.1916 (5)	0.99482 (14)	0.2323 (3)	0.0539 (8)	
H4	0.3230	1.0028	0.2029	0.065*	
C5	0.1695 (4)	0.95122 (13)	0.3364 (3)	0.0414 (7)	
C6	−0.0285 (4)	0.93888 (12)	0.3764 (3)	0.0367 (6)	
C7	−0.1427 (4)	0.85935 (13)	0.5476 (3)	0.0448 (7)	
H7A	−0.2662	0.8860	0.5547	0.054*	
H7B	−0.0790	0.8493	0.6391	0.054*	
C8	−0.2120 (4)	0.79717 (13)	0.4733 (3)	0.0435 (7)	
H8A	−0.3149	0.7754	0.5220	0.052*	
H8B	−0.2801	0.8074	0.3830	0.052*	
C9	−0.0301 (4)	0.75382 (12)	0.4618 (2)	0.0344 (6)	
C10	0.4117 (5)	0.65844 (13)	0.5972 (3)	0.0482 (7)	
H10	0.4180	0.6501	0.6899	0.058*	
C11	0.5649 (5)	0.63636 (13)	0.5222 (3)	0.0526 (8)	
H11	0.6778	0.6127	0.5648	0.063*	
C12	0.5546 (5)	0.64870 (13)	0.3839 (3)	0.0498 (8)	
H12	0.6615	0.6333	0.3361	0.060*	
C13	0.3915 (4)	0.68287 (13)	0.3161 (3)	0.0438 (7)	
H13	0.3856	0.6904	0.2232	0.053*	
C14	0.2348 (4)	0.70614 (12)	0.3900 (2)	0.0344 (6)	
C15	0.2474 (4)	0.69362 (12)	0.5293 (2)	0.0363 (6)	
N1	0.0085 (3)	0.89470 (10)	0.4772 (2)	0.0388 (6)	
N2	0.2169 (4)	0.87999 (11)	0.4968 (2)	0.0491 (6)	
N3	0.3165 (3)	0.91386 (12)	0.4137 (3)	0.0530 (7)	
N4	0.0740 (3)	0.72406 (10)	0.5709 (2)	0.0398 (6)	
H14	0.0439	0.7286	0.6512	0.060*	
N5	0.0578 (3)	0.74388 (10)	0.3498 (2)	0.0379 (5)	

### Atomic displacement parameters (Å^2^)

**Table:**

	*U*^11^	*U*^22^	*U*^33^	*U*^12^	*U*^13^	*U*^23^
C1	0.0375 (16)	0.0444 (17)	0.0615 (19)	−0.0022 (14)	0.0082 (14)	−0.0019 (15)
C2	0.0480 (19)	0.0470 (19)	0.074 (2)	0.0052 (15)	−0.0048 (16)	0.0073 (18)
C3	0.060 (2)	0.0481 (18)	0.061 (2)	−0.0064 (16)	0.0035 (17)	0.0156 (16)
C4	0.0446 (18)	0.0541 (19)	0.065 (2)	−0.0064 (15)	0.0167 (16)	0.0090 (17)
C5	0.0365 (15)	0.0413 (16)	0.0473 (17)	−0.0015 (13)	0.0086 (13)	−0.0024 (14)
C6	0.0373 (16)	0.0330 (15)	0.0399 (16)	−0.0013 (12)	0.0045 (13)	−0.0062 (13)
C7	0.0474 (17)	0.0458 (17)	0.0440 (17)	−0.0007 (14)	0.0173 (14)	−0.0020 (14)
C8	0.0403 (16)	0.0504 (17)	0.0411 (16)	−0.0085 (14)	0.0103 (13)	−0.0003 (14)
C9	0.0388 (15)	0.0400 (15)	0.0247 (14)	−0.0109 (12)	0.0045 (12)	−0.0014 (12)
C10	0.0574 (19)	0.0471 (17)	0.0390 (16)	−0.0050 (15)	0.0007 (15)	0.0113 (14)
C11	0.0498 (19)	0.0397 (17)	0.067 (2)	0.0042 (14)	0.0008 (16)	0.0082 (16)
C12	0.0539 (19)	0.0389 (17)	0.059 (2)	0.0011 (15)	0.0152 (16)	−0.0045 (15)
C13	0.0539 (18)	0.0435 (17)	0.0349 (15)	−0.0027 (14)	0.0092 (14)	−0.0063 (13)
C14	0.0454 (16)	0.0332 (14)	0.0249 (13)	−0.0068 (13)	0.0057 (12)	−0.0048 (12)
C15	0.0455 (16)	0.0321 (15)	0.0317 (14)	−0.0060 (13)	0.0063 (13)	−0.0012 (12)
N1	0.0346 (13)	0.0403 (13)	0.0422 (13)	−0.0008 (10)	0.0072 (10)	0.0007 (11)
N2	0.0369 (14)	0.0555 (16)	0.0547 (15)	0.0026 (12)	0.0045 (12)	0.0074 (13)
N3	0.0338 (13)	0.0617 (16)	0.0645 (17)	−0.0004 (12)	0.0099 (12)	0.0116 (14)
N4	0.0486 (14)	0.0480 (14)	0.0240 (12)	−0.0074 (11)	0.0095 (10)	−0.0008 (10)
N5	0.0419 (13)	0.0465 (13)	0.0257 (11)	−0.0022 (11)	0.0057 (10)	0.0004 (10)

### Geometric parameters (Å, °)

**Table:**

C1—C2	1.370 (4)	C8—H8B	0.9700
C1—C6	1.388 (4)	C9—N5	1.316 (3)
C1—H1	0.9300	C9—N4	1.348 (3)
C2—C3	1.393 (4)	C10—C11	1.372 (4)
C2—H2	0.9300	C10—C15	1.383 (4)
C3—C4	1.353 (4)	C10—H10	0.9300
C3—H3	0.9300	C11—C12	1.387 (4)
C4—C5	1.394 (4)	C11—H11	0.9300
C4—H4	0.9300	C12—C13	1.366 (4)
C5—N3	1.376 (3)	C12—H12	0.9300
C5—C6	1.386 (3)	C13—C14	1.391 (3)
C6—N1	1.357 (3)	C13—H13	0.9300
C7—N1	1.453 (3)	C14—N5	1.391 (3)
C7—C8	1.529 (4)	C14—C15	1.397 (3)
C7—H7A	0.9700	C15—N4	1.375 (3)
C7—H7B	0.9700	N1—N2	1.350 (3)
C8—C9	1.481 (4)	N2—N3	1.303 (3)
C8—H8A	0.9700	N4—H14	0.8453
			
C2—C1—C6	116.0 (3)	N5—C9—N4	112.7 (2)
C2—C1—H1	122.0	N5—C9—C8	125.0 (2)
C6—C1—H1	122.0	N4—C9—C8	122.1 (2)
C1—C2—C3	122.0 (3)	C11—C10—C15	117.2 (3)
C1—C2—H2	119.0	C11—C10—H10	121.4
C3—C2—H2	119.0	C15—C10—H10	121.4
C4—C3—C2	121.9 (3)	C10—C11—C12	121.3 (3)
C4—C3—H3	119.0	C10—C11—H11	119.4
C2—C3—H3	119.0	C12—C11—H11	119.4
C3—C4—C5	117.3 (3)	C13—C12—C11	121.8 (3)
C3—C4—H4	121.3	C13—C12—H12	119.1
C5—C4—H4	121.3	C11—C12—H12	119.1
N3—C5—C6	108.4 (2)	C12—C13—C14	118.1 (3)
N3—C5—C4	131.2 (3)	C12—C13—H13	120.9
C6—C5—C4	120.4 (3)	C14—C13—H13	120.9
N1—C6—C5	104.6 (2)	C13—C14—N5	130.6 (2)
N1—C6—C1	133.1 (2)	C13—C14—C15	119.6 (2)
C5—C6—C1	122.3 (3)	N5—C14—C15	109.8 (2)
N1—C7—C8	111.6 (2)	N4—C15—C10	133.1 (2)
N1—C7—H7A	109.3	N4—C15—C14	104.8 (2)
C8—C7—H7A	109.3	C10—C15—C14	122.1 (3)
N1—C7—H7B	109.3	N2—N1—C6	109.9 (2)
C8—C7—H7B	109.3	N2—N1—C7	120.6 (2)
H7A—C7—H7B	108.0	C6—N1—C7	129.0 (2)
C9—C8—C7	111.8 (2)	N3—N2—N1	109.1 (2)
C9—C8—H8A	109.3	N2—N3—C5	108.0 (2)
C7—C8—H8A	109.3	C9—N4—C15	107.9 (2)
C9—C8—H8B	109.3	C9—N4—H14	124.0
C7—C8—H8B	109.3	C15—N4—H14	127.7
H8A—C8—H8B	107.9	C9—N5—C14	104.9 (2)
			
C6—C1—C2—C3	−0.5 (4)	N5—C14—C15—N4	0.5 (3)
C1—C2—C3—C4	0.5 (5)	C13—C14—C15—C10	−0.1 (4)
C2—C3—C4—C5	−0.9 (5)	N5—C14—C15—C10	−178.3 (2)
C3—C4—C5—N3	−179.5 (3)	C5—C6—N1—N2	−0.8 (3)
C3—C4—C5—C6	1.5 (4)	C1—C6—N1—N2	−179.3 (3)
N3—C5—C6—N1	0.4 (3)	C5—C6—N1—C7	−173.1 (2)
C4—C5—C6—N1	179.6 (2)	C1—C6—N1—C7	8.4 (5)
N3—C5—C6—C1	179.1 (2)	C8—C7—N1—N2	−83.6 (3)
C4—C5—C6—C1	−1.7 (4)	C8—C7—N1—C6	88.0 (3)
C2—C1—C6—N1	179.4 (3)	C6—N1—N2—N3	1.0 (3)
C2—C1—C6—C5	1.2 (4)	C7—N1—N2—N3	174.1 (2)
N1—C7—C8—C9	60.1 (3)	N1—N2—N3—C5	−0.7 (3)
C7—C8—C9—N5	−105.2 (3)	C6—C5—N3—N2	0.2 (3)
C7—C8—C9—N4	69.2 (3)	C4—C5—N3—N2	−178.9 (3)
C15—C10—C11—C12	−0.2 (4)	N5—C9—N4—C15	1.9 (3)
C10—C11—C12—C13	−0.4 (4)	C8—C9—N4—C15	−173.2 (2)
C11—C12—C13—C14	0.8 (4)	C10—C15—N4—C9	177.2 (3)
C12—C13—C14—N5	177.2 (3)	C14—C15—N4—C9	−1.4 (3)
C12—C13—C14—C15	−0.5 (4)	N4—C9—N5—C14	−1.5 (3)
C11—C10—C15—N4	−177.9 (3)	C8—C9—N5—C14	173.4 (2)
C11—C10—C15—C14	0.5 (4)	C13—C14—N5—C9	−177.3 (3)
C13—C14—C15—N4	178.7 (2)	C15—C14—N5—C9	0.6 (3)

### Hydrogen-bond geometry (Å, °)

**Table:**

*D*—H···*A*	*D*—H	H···*A*	*D*···*A*	*D*—H···*A*
N4—H14···N5^i^	0.84	2.04	2.855 (3)	162
C1—H1···N3^ii^	0.93	2.54	3.456 (3)	167

Symmetry codes: (i) *x*, −*y*+3/2, *z*+1/2; (ii) *x*−1, *y*, *z*.
